# Special histological subtypes of breast cancer in a Hispanic Latino population

**DOI:** 10.1371/journal.pone.0333139

**Published:** 2025-10-03

**Authors:** Gonzalo Ziegler Rodriguez, Gabriel De La Cruz Ku, Alanna Hickey, Sarah Roberts, Sheila Katherine Diaz-Mora, Augusto Ordonez, Luis Piedra Delgado, Jiddu Guart, Juan Haro Varas, Jorge Dunstan Yataco, Jose Manuel Cotrina Concha

**Affiliations:** 1 Department of Breast, Skin and Soft Tissue Surgical Oncology, Instituto Nacional de Enfermedades Neoplasicas (INEN), Lima, Peru,; 2 Chief, Breast Surgery Unit, Clinica Ziegler, Lima, Peru,; 3 Universidad Cientifica del Sur, Lima, Peru; 4 University of Massachusetts Medical School, Worcester, Massachusetts, United States of America; 5 Department of Pathology, Instituto Nacional de Enfermedades Neoplasicas, Lima, Peru; 6 Department of Surgical Oncology, Instituto Nacional de Enfermedades Neoplasicas, Lima, Peru; 7 Department of Medical Oncology, Instituto Nacional de Enfermedades Neoplasicas, Lima, Peru; OMICS, PERU

## Abstract

**Introduction:**

Special histologic subtypes of breast cancer have a unique biological behavior and outcomes. The literature has demonstrated that histologic and phenotype subclassification of breast cancer varies according to race and populations. Our aim was to describe the clinicopathological characteristics and outcomes of breast cancer with special histological subtypes in a Latin/Hispanic population.

**Methods:**

A retrospective study was conducted. We reviewed the medical records of patients newly diagnosed with special histological subtypes of breast cancer at a single tertiary reference cancer center in Peru from 2014 to 2019.

**Results:**

A total of 479 patients were included, the median age at diagnosis was 55 years (range 26–89). The majority of patients were from a metropolitan area (59.1%). The most common histological subtype was lobular (34.9%), followed by mucinous (12.7%), papillary (12.5%), apocrine (6.9%), metaplastic (5.4%), medullary (3.8%), cribriform (3.3%), neuroendocrine (0.8%), and 9.2% mixed histology. 61.6% had a moderately differentiated grade. The most common phenotype at diagnosis was HR + HER2- (57.7%), followed by triple-negative breast cancer (TNBC)(13.2%), showing a similar pattern after neoadjuvant therapy (NAT); HR + HER- (61.7%), and TNBC (16.3%). At diagnosis most patients were stage T2 (40.3%), N0 (61.0%) and stage II (40.7%); while after NAT, stage I (64.7%) was the most common. In regard to NAT, 45.9% received chemotherapy, 31.5% hormone therapy, 15.7% trastuzumab, and 5.8% radiotherapy. The majority underwent mastectomy (71.4%). In relation to adjuvant treatment, 72.7% received chemotherapy, 74.1% hormone therapy, 9.4% trastuzumab, and 76.4% radiotherapy. Loco-regional and distant recurrence occurred in 4.2% and 12.7%, respectively. With a median follow-up of 97 months (8 years), the overall survival (OS) at 5 years was 82%, with patients with cribriform histology presenting the best rate (100%) compared to the worst observed in patients with metaplastic histology (54%).

**Conclusions:**

The most frequent special histologic subtypes of breast cancer in Latino-Hispanic patients were lobular, mucinous, papillary, metaplastic, and apocrine carcinomas. Patients were diagnosed at more advanced stages and more frequently presented a TNBC phenotype compared to the non-Hispanic White population. Certain histological subtypes in our population presented worse OS rates.

## Introduction

Breast cancer is the most common cancer diagnosis worldwide. In countries with a high human development index, 1 in 12 women will be diagnosed during their lifetime in contrast to 1 in 27 women in countries with a low human development index [1]. Stage at diagnosis and a predominant proportion of histologic subtypes and phenotypes vary significantly by region and race across the world [2]. For instance, on a global scale, a higher stage at diagnosis is observed in countries with a lower socioeconomic status, having serious implications on the overall prognosis of these patients. A meta-analysis found that the rate of metastatic breast cancer at initial diagnosis ranged from 0.0–6.0% in North America compared to 5.4–23.7% in Central and South American countries [2].

The differences in incidence and mortality by breast cancer cannot only be explained by global borders or social factors but rather crucial differences based on ethnic background have an impact on tumor microenvironment and biology [3,4]. For example, Black women present a higher incidence and mortality of triple-negative breast cancer (TNBC) compared to other races/ethnicities [4]. Regarding histology and phenotype, differences in the rates of both based on ethnicity have also been described [5]. A US-based epidemiologic study of breast cancer histology and phenotype in relation to ethnicity revealed higher rates of TNBC and HER2 + phenotypes in Black patients compared to non-Hispanic White patients (NHW), as well as a lower frequency of grade 1 and 2 histology in Asian/Pacific Islanders versus NHW patients.

Invasive ductal carcinoma is the most common breast cancer histology [6] and 25% of breast cancers are classified as special histologic subtypes [2,7]. According to the World Health Organization (WHO) special histologic subtypes are defined as invasive breast carcinomas with distinct architectural and structural patterns which differ from the common invasive carcinoma of no special type [8]. They have a distinct biological behavior in terms of prognosis, metastatic potential, treatment response, and outcomes and represent potential targets for therapy [8,9]. For example, tubular carcinoma has been associated with a favorable prognosis and is more frequently seen in White compared to Black patients [10]. In contrast, the metaplastic carcinoma subtype has a poorer prognosis in both races. Histologic subtypes also vary according to race and region. Higher proportions of dominant subtypes of lobular carcinoma and tubular adenocarcinoma are found in NHW compared to Hispanic White counterparts [5].

Although the clinical and biological heterogenicity of special histologic subtypes have been described, most of the literature published in this regard is from North American, European, or Asian cohorts, leaving a significant gap in knowledge of Hispanic Latin American populations. Regional and ethnic variation may influence the distribution of these subtypes as well as the sociodemographic characteristics, clinical behavior, phenotypes, therapeutic response, and survival outcomes. Moreover, these subtypes are usually excluded from clinical trials, thereby limiting the evidence to formulate guidelines in real-world practice. To date, data on the epidemiology, treatment, and outcomes of special histologic subtypes of breast cancer in Hispanic Latino populations are scarce. Therefore, to address this critical gap and contribute novel data on this topic, we sought to specifically describe and analyze the clinicopathological, therapeutic characteristics and outcomes of special subtypes of breast cancer in a Latin/Hispanic population.

## Materials and methods

### Study design and population

A retrospective cohort study was conducted. Medical records of patients diagnosed with breast cancer and treated at the “Instituto Nacional de Enfermedades Neoplasicias” (INEN), Lima, Peru, from January 1^st^, 2014 to December 31^st^, 2019 were reviewed. The patients were followed until October 2024. The INEN is a tertiary referral cancer center located in Lima, the capital of Peru. All patients treated at this institution are under the coverage of the Ministry of Health, which is the largest insurance network in Peru. Medical records were accessed for research purposes from 15-08-2023–15-12-2024. All the data were collected manually. Inclusion criteria were: patients 18 years and older with a confirmed pathological diagnosis of special or rare histological subtypes of breast cancer, which are defined as subtypes beyond invasive ductal carcinoma [11]; American Joint Committee on Cancer (AJCC) stages I to IV [12]; all specimens were reviewed by the Pathology Department of the Institute. Each diagnosis was independently reviewed and confirmed by at least two attending pathologists from the Breast Pathology Group of the institute. Final classification was achieved by consensus following the criteria outlined in the WHO Classification of Tumours of the Breast, 5^th^ edition [8]. The histopathological results of the samples were reported in the same manner throughout the study period with no major changes. Exclusion criteria included patients lost to follow-up and incomplete medical records. All patients were treated according to the National Comprehensive Cancer Network guidelines.It should be noted that trastuzumab has been available in Peru since 2019 and pertuzumab since 2023, however no other immunotherapy regimens nor checkpoint inhibitors are available for HER2 positive patients or those with TNBC.

### Variables

The type of residence was classified according to the United States Department of Agriculture guidelines [13]. Metropolitan areas were defined as regions with central counties containing urban areas of 50,000 people or more, while non-metropolitan areas refer to less accessible regions without urban centers. The special histologic subtypes included were based on the WHO classification system [8]: lobular, cribriform, apocrine, metaplastic, mucinous, neuroendocrine, medullary, papillary, micropapillary, tubular, mucoepidermoid, adenoid cystic, clear cell, and cystoadenocarcinoma. Mixed subtypes were defined as tumors containing two or more histological patterns, with at least one component being a special histologic subtype. All the histologic subtypes available during the study period were included to obtain a more accurate characterization of Hispanic Latino population.

Phenotypes were classified according to estrogen-receptor (ER), progesterone-receptor (PR), and human epidermal growth factor receptor 2 (HER2) expression. If HER2 was inconclusive, the patients underwent evaluation with fluorescence *in situ* hybridization (FISH) to confirm the positivity or negative of the result with a cut-off of two. The TNBC phenotype was defined as ER negative or <1%, PR negative or <1%, and HER2 negative. Genetic tests such as BRCA1/2 are not covered by the insurance, therefore these results were not available. All patients were classified according to the 8^th^ edition of the AJCC and histologic subtype. Event-free survival (EFS) was defined as, the time from the date of surgery until locoregional or distant relapse in the patients who underwent surgery and as the time from diagnosis to disease progression, death or the end of the study, whichever occurred first, in patients who did not undergo surgery. Overall survival (OS) was defined as the time from diagnosis of the primary tumor until death or end of the study. Recurrence was confirmed with biopsy after clinical examination, ultrasound, computed tomography (CT) scan, magnetic resonance imaging, or positron emission tomography- computed tomography was performed.

### Statistical analysis

No sample size was calculated. All the patients fulfilling the inclusion and exclusion criteria during the study period were included. Descriptive statistics were used to describe the sociodemographic, clinical, and therapeutic characteristics of the patients. Quantitative variables were described with mean and standard deviation, or median with interquartile range (IQR), depending on the normality of the variable. Qualitative variables were described with frequencies and percentages. Analysis of variance was used to assess the relationship between qualitative and quantitative variables, while chi-square or Fisher’s exact tests were used to assess the relationship between qualitative variables. Survival rates were calculated using Kaplan-Meier curves, and the Log-rank test was used to assess the differences in survival rates between different groups at 3 and 5 years of follow-up. Patients lost to follow-up were excluded from the analysis. Variables with a frequency less than four were not included in bivariate and survival analyses. Missing data for key variables, such as Ki-67, phenotype, and staging, are described in the tables. These cases were not included in the statistical analyses and no imputation was performed. Prognostic factors were calculated using Cox-regression analysis, risk of death or recurrence was expressed with hazard ratios, with 95% confidence intervals. To address any potential bias in our analysis, all the population fulfilling the eligibility criteria was included in our analysis, no sample was calculated. The Cox proportional hazard assumption was met by the goodness-of-fit-test. A p-value <0.05 was considered statistically significant. The Package for Social Sciences software version 28.0 was used for the analysis.

### Ethics

This study was approved by the Institutional Review Board (IRB) of INEN (INEN 23–41). Consent was waived by the Ethics Committee due to the retrospective design of our study. Data was collected confidentially by the principal investigator after obtaining authorization to access the medical records by the IRB. The study was self-funded by the authors, with no financial support being received from any institution. All data was managed with strict confidentiality and only used for study purposes; it was de-identified and clinical information was codified (S1 File).

## Results

**Fig 1:**
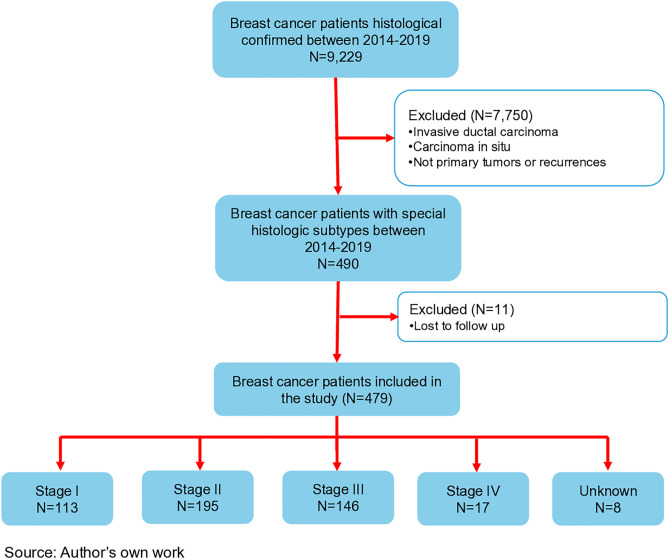
Graphic schematization of patients inclusion in the study.

Out of a total of 8,229 patients with breast cancer, 490 were diagnosed with special histologic subtypes of breast cancer, and of these 479 patients met the eligibility criteria (Fig 1). The mean age at diagnosis was 55.59 years (IQR 46–65.75 years), and the majority of the population lived at a metropolitan area (59.1%). The most common histologic subtype was invasive lobular carcinoma (34.9%), followed by mucinous (12.&%), papillary (12.5%), apocrine (6.9%), metaplastic (5.4%), medullary (3.8%), and cribriform (3.3%). Most of the tumors were intermediate grade (63.6%) with high Ki76 (72.3%). The most common phenotype was hormone receptor (HR) +/ HER2 – (57.7%), followed by TNBC (15.7%), HR + HER2+ (8.4%), and HR-HER2+ (5.0%). Most of patients were diagnosed as T2 (40.5) and T1 (25.8%), N0 (61.3%) and N1 (24.6%), M0 (96.0%); and stage II (41.4%) and stage III (31.0%) (Table 1).

**Table 1:** Demographic and clinicopathological characteristics of Latin-Hispanic patients with special histological subtypes of breast cancer at diagnosis.

Characteristics	N/mean (N = 479)	Percentage (%)/SD
Age – years, mean	55.59	12.30
Area of residence		
Metropolitan areas	283	59.1
Non-metropolitan area	196	40.9
Histology subtype		
Invasive lobular carcinoma	167	34.9
Mucinous	61	12.7
Papillary	60	12.5
Apocrine	33	6.9
Metaplastic	26	5.4
Tubular	21	4.4
Micropapillary	20	4.2
Medullary	18	3.8
Cribriform	16	3.3
Neuroendocrine	4	0.8
Clear cell	3	0.6
Adenoid cystic	3	0.6
Mucoepidermoid	2	0.4
Cystoadenocarcinoma	1	0.2
Mixed	44	9.2
Grade		
Low	53	11.4
Intermediate	295	63.6
High	116	25.0
Missing	15	
Initial Phenotype		
HR + HER2-	276	57.7
HR + HER2+	40	8.4
HR- HER2+	24	5.0
Triple-negative	75	15.7
HR + HER2 indeterminate	63	13.2
Missing	1	
Initial Ki67, %		
≤20	130	27.7
>20	340	72.3
Missing	9	
Initial T Stage		
0	1	0.2
1	123	25.8
2	193	40.5
3	89	18.7
4	71	14.9
Missing	2	
Initial N Stage		
0	292	61.3
1	117	24.6
2	46	9.7
3	21	4.4
Missing	3	
Initial M stage		
0	456	96.0
1	19	4.0
Missing	4	
Initial AJCC Stage		
I	113	24.0
II	195	41.4
III	146	31.0
IV	17	3.6
Missing	8	

Source: Authors’ own work.

SD: standard deviation; AJCC: American Joint Committee on Cancer

Regarding neoadjuvant therapies, around half of the population received neoadjuvant chemotherapy (48.6%); 31.5%, hormonal therapy; 15.7%, trastuzumab; 5.8%, radiotherapy. Breast conserving surgery was performed in 28.6% of patients. Most of the patients received adjuvant therapies, 72.7%, chemotherapy; 76.4%, radiotherapy; 74.1%, hormonal therapy; and 9.4%, trastuzumab (Table 2). When assessing the pathologic characteristics after neoadjuvant therapies, 66.1% were classified as Stage I, followed by stage III (16.2%) and stage II (14.1%). The most common phenotype was HR + HER2- (61.7%) followed by TNBC (16.3%) (Table 3).

**Table 2:** Therapeutic characteristics of Latin-Hispanic patients with special histological subtypes of breast cancer.

Characteristics	N/mean (N = 479)	Percentage (%)
Neoadjuvant chemotherapy		
No	246	51.4
Yes	233	48.6
Neoadjuvant radiotherapy		
No	451	94.2
Yes	28	5.8
Neoadjuvant hormonal therapy		
No	328	68.5
Yes	151	31.5
Neoadjuvant trastuzumab		
No	404	84.3
Yes	75	15.7
Surgery		
Breast conserving surgery	132	71.4
Mastectomy	137	28.6
Missing		
Adjuvant chemotherapy		
No	131	27.3
Yes	348	72.7
Adjuvant radiotherapy		
No	113	23.6
Yes	366	76.4
Adjuvant hormonal therapy		
No	124	25.8
Yes	355	74.1
Adjuvant trastuzumab		
No	434	90.6
Yes	45	9.4

Source: Authors’ own work.

**Table 3:** Pathologic characteristics and outcomes of Latin-Hispanic patients with special histological subtypes of breast cancer after neoadjuvant treatment.

Characteristics	N/mean (N = 479)	Percentage (%)
Final AJCC Stage		
I	310	66.1
II	66	14.1
III	76	16.2
IV	17	3.6
Missing	10	
Final Phenotype		
HR + HER2-	295	61.7
HR + HER2+	56	11.7
HR- HER2+	25	5.2
Triple negative	78	16.3
HR + HER2 indeterminate	24	5.0
Missing	1	
Final Ki67, %		
≤20	127	26.9
>20	345	73.1
Missing	7	
Locoregional recurrence		
No	459	95.8
Yes	20	4.2
Distant recurrence		
No	418	87.3
Yes	61	12.7
Metastasis		
Bone	21	4.4
Multiple solid organs	10	2.1
Oligometastatic to solid organ	30	6.3

Source: Authors’ own work. AJCC: American Joint Committee on Cancer

Subgroup analysis was performed according to subtypes. Most of the patients with metaplastic (57.7%) and medullary (61.1%) histology lived in non-metropolitan areas (p = 0.010). Among the metaplastic tumors, 92.3% were high grade and 80.8% of these were TNBC, followed by medullary tumors (83.3%, p < 0.001). A Ki67 > 20% was more frequently present in the medullary (94.4%), apocrine (90.9%), and metaplastic (84.6%) subtypes (p < 0.001). Advanced T stages (T3-4) were related to metaplastic (65.4%), apocrine (63.6%), and medullary (61.1%) subtypes (p < 0.001); while advanced N stages were related to apocrine (57.6%), mixed (55.8%), and metaplastic (46.2%) subtypes (p = 0.037). In terms of initial AJCC staging, the subtypes related to stage III were apocrine (57.6%), metaplastic (50.0%), and medullary (44.4%, p = 0.008) (Table 4 and S1 Table).

**Table 4:** Demographic and clinicopathological characteristics of Latin-Hispanic patients with breast cancer at diagnosis according to special histologic subtype.

Characteristics	Lobular N = 167	Cribriform N = 16	Apocrine N = 33	Metaplastic N = 26	Mucinous N = 61	Neuroendocrine N = 4	Medullary N = 18	Papillary N = 60	Micropapillary N = 20	Tubular N = 21	Mixed 44	P value
Age – years, mean ± SD	51.08 ± 12.44	60.06 ± 9.98	60.44 ± 12.45	50.64 ± 9.10	60.35 ± 11.67	55.25 ± 9.29	58.39 ± 11.84	61.35 ± 10.34	55.70 ± 10.73	56.71 ± 12.64	52.86 ± 11.00	<0.001^a^
Area of residence												0.013 ^b^
Metropolitan areas	96 (57.5)	11 (68.8)	19 (57.6)	11 (42.3)	37 (60.7)	0 (0)	7 (38.9)	41 (68.3)	17 (85.0)	15 (71.4)	23 (52.3)	
Non-metropolitan area	71 (42.5)	5 (31.3)	14 (42.4)	15 (57.7)	24 (39.3)	4 (100.0)	11 (61.1)	19 (31.7)	3 (15.0)	6 (28.6)	21 (47.7)	
Grade												
Low – Intermediate	131 (81.4)	15 (100.0)	22 (66.7)	2 (7.7)	54 (90.0)	2 (50.0)	10 (58.8)	42 (76.4)	17 (89.5)	20 (95.2)	28 (63.6)	<0.001 ^c^
High	30 (18.6)	0 (0.0)	11 (33.3)	24 (92.3)	6 (10.0)	2 (50.0)	7 (41.2)	13 (23.6)	2 (10.5)	1 (4.8)	16 (36.4)	
Initial Phenotype												
HR + HER2-	133 (79.6)	8 (50.0)	9 (27.3)	2 (7.7)	52 (86.7)	1 (25.0)	0 (0.0)	30 (50.0)	7 (35.0)	14 (66.7)	20 (45.5)	<0.001 ^c^
HR + HER2+	5 (3.0)	1 (6.3)	5 (15.2)	1 (3.8)	1 (1.7)	0 (0.0)	2 (11.1)	14 (23.3)	4 (20.0)	0 (0.0)	7 (15.9)	
HR- HER2+	2 (1.2)	5 (31.3)	5 (15.2)	0 (0.0)	0 (0.0)	0 (0.0)	1 (5.6)	4 (6.7)	2 (10.0)	0 (9.5)	2 (4.5)	
Triple negative	9 (5.4)	1 (6.3)	6 (18.2)	21 (80.8)	1 (1.7)	2 (50.0)	15 (83.3)	6 (10.0)	1 (5.0)	1 (4.8)	6 (13.6)	
HR + HER2 indeterminate	18 (10.8)	1 (6.3)	8 (24.2)	2 (7.7)	6 (10.0)	1 (25.0)	0 (0.0)	6 (10.0)	6 (30.0)	4 (19.0)	9 (20.5)	
Initial Ki67, > 20%	110 (66.7)	11 (68.8)	30 (90.9)	22 (84.6)	30 (49.2)	4 (100.0)	17 (94.4)	42 (76.4)	16 (84.2)	16 (76.2)	34 (79.1)	<0.001 ^c^
Initial AJCC Stage												
I-II	118 (71.1)	12 (75.0)	12 (36.4)	10 (38.5)	46 (75.4)	2 (50.0)	9 (50.0)	39 (69.6)	10 (50.0)	19 (95.0)	26 (61.9)	0.008 ^c^
III	43 (25.9)	4 (25.0)	19 (57.6)	13 (50.0)	15 (24.6)	2 (50.0)	8 (44.4)	15 (26.8)	8 (40.0)	1 (5.0)	15 (35.7)	
IV	5 (3.0)	0 (0.0)	2 (6.1)	3 (11.5)	0 (0.0)	0 (0.0)	1 (5.6)	2 (3.6)	2 (10.0)	0 (0.0)	1 (2.4)	

^a^Analysis of variance

^b^chi-square test

^c^Fisher’s exact test

AJCC: American Joint Committee on Cancer

Source: Authors’ own work.

Further analysis of the therapeutic characteristics according to subtypes showed that the subtypes that more frequently underwent neoadjuvant chemotherapy were the apocrine (84.8%) and medullary (83.3%) subtypes, while neoadjuvant radiotherapy was administered in the metaplastic subtype (15.4%), neoadjuvant hormonal therapy was received by patients with papillary (46.7%) and apocrine subtypes (42.4%), and neoadjuvant treatment with trastuzumab was given to patients with an apocrine subtype (48.5%) (p < 0.05). Moreover, the patients who most frequently underwent mastectomy were those with metaplastic (100.0%) and apocrine (87.9%) histology. In terms of adjuvant therapies, adjuvant chemotherapy was related to medullary (94.4%), and apocrine subtypes (93.9%); adjuvant radiotherapy to cribriform (93.8%) and mixed subtypes (90.9%); adjuvant hormonal therapy to mucinous (98.4%) and lobular subtypes (91.0%); and adjuvant trastuzumab to patients with apocrine (33.3%) and cribriform subtypes (31.3%) (Table 5).

**Table 5:** Pathologic characteristics and outcomes of Latin-Hispanic patients with breast cancer after neoadjuvant treatment according to special histological subtypes.

Characteristics	Lobular N = 167	Cribriform N = 16	Apocrine N = 33	Metaplastic N = 26	Mucinous N = 61	Neuroendocrine N = 4	Medullary N = 18	Papillary N = 60	Micropapillary N = 20	Tubular N = 21	Mixed 44	P value
Final AJCC Stage												
I-II	145 (87.3)	16 (100.0)	21 (63.6)	9 (37.5)	52 (85.2)	2 (50.0)	10 (58.8)	47 (83.9)	13 (65.0)	21 (100.0)	35 (83.3)	<0.001^a^
III	16 (9.6)	0 (0.0)	10 (30.3)	12 (50.0)	9 (14.8)	2 (50.0)	6 (35.3)	7 (12.5)	5 (25.0)	0 (0.0)	6 (14.3)	
IV	5 (3.0)	0 (0.0)	2 (6.1)	3 (12.5)	0 (0.0)	0 (0.0)	1 (5.9)	2 (3.6)	2 (10.0)	0 (0.0)	1 (2.4)	
Final Phenotype												
HR + HER2-	139 (83.2)	6 (37.5)	10 (30.3)	2 (7.7)	57 (93.4)	1 (25.0)	1 (5.6)	32 (54.2)	9 (45.0)	16 (76.2)	21 (47.7)	<0.001^b^
HR + HER2+	7 (4.2)	2 (12.5)	7 (21.2)	1 (3.8)	1 (1.6)	0 (0.0)	2 (11.1)	13 (22.0)	8 (40.0)	2 (9.5)	12 (27.3)	
HR- HER2+	2 (1.2)	5 (31.3)	6 (18.2)	0 (0.0)	0 (0.0)	1 (25.0)	1 (5.6)	5 (8.5)	1 (5.0)	2 (9.5)	2 (4.5)	
Triple negative	11 (6.6)	2 (12.5)	6 (18.2)	22 (84.6)	0 (0.0)	2 (50.0)	14 (77.8)	5 (8.5)	2 (10.0)	1 (4.8)	6 (13.6)	
HR + HER2 indeterminate	8 (4.8)	1 (6.3)	4 (12.1)	1 (3.8)	3 (4.9)	0 (0.0)	0 (0.0)	4 (6.8)	0 (0.0)	0 (0.0)	3 (6.8)	
Final Ki67 > 20%	108 (65.5)	13 (81.3)	29 (87.9)	24 (92.3)	33 (54.1)	4 (100.0)	17 (94.4)	44 (78.6)	14 (73.7)	18 (85.7)	33 (75.0)	<0.001^b^
Locoregional recurrence	2 (1.2)	0 (0.0)	1 (3.0)	4 (15.4)	3 (4.9)	0 (0.0)	2 (11.1)	1 (1.7)	2 (10.0)	1 (4.8)	3 (6.8)	0.0239^b^
Distant recurrence	33 (19.8)	1 (6.3)	4 (12.1)	0 (0.0)	5 (8.2)	2 (50.0)	4 (22.2)	2 (3.3)	4 (20.0)	0 (0.0)	5 (11.4)	0.002^b^
Metastasis												
Bone	15 (9.0)	0 (0.0)	1 (3.0)	0 (0.0)	1 (1.6)	1 (25.0)	1 (5.6)	0 (0.0)	2 (10.0)	0 (0.0)	0 (0.0)	0.013^b^
Multiple solid organs	6 (3.6)	0 (0.0)	1 (3.0)	0 (0.0)	0 (0.0)	0 (0.0)	1 (5.6)	0 (0.0)	0 (0.0)	0 (0.0)	1 (2.3)	0.621^b^
Oligometastatic to solid organ	12 (7.2)	1 (6.3)	2 (6.1)	0 (0.0)	4 (6.6)	1 (25.0)	2 (11.1)	2 (3.3)	2 (10.0)	0 (0.0)	4 (9.1)	0.600^b^

^a^chi-square test

^b^Fisher’s exact test

AJCC: American Joint Committee on Cancer

Source: Authors’ own work.

Subgroup analysis regarding pathologic characteristics and outcomes after neoadjuvant therapies according to the histological subtype revealed that the subtypes related to early stages were cribriform (100.0%) and mucinous (85.2%) (p < 0.001), while the subtypes with the highest rates of locoregional and distant recurrence had metaplastic (15.4%) and neuroendocrine histology (50.0%), respectively (p < 0.05). No differences were found in multiple or oligometastasis to solid organs among the histologic subtypes (S1 Table).

In terms of outcomes, locoregional and distant recurrence was present in 4.2% and 12.7% of patients, respectively. The most common distant metastasis was visceral oligometastasis (6.3%), followed by bone (4.4%), and multiple visceral metastases (2.1%) (Table 3). With a median follow-up of 97.7 months (8.2 years), the histologic subtype with the best OS at 5 years of follow-up was cribriform (100%); however, this subgroup included only 16 patients, therefore these findings should be interpreted with caution. Other subtypes demonstrated the following 5-year OS rates: papillary (90.0%), mixed (88.6%), mucinous (88.3%), lobular (78.9%), and apocrine (78.8%) (p = 0.001) (Table 6, ) and similar 5-year EFS rates, cribriform (100%), papillary (88.9%), mixed (87.6%), mucinous (86.1%), lobular (76.7%), and apocrine (75.8%) (p < 0.001) (Table 4, Fig 5). The phenotype with the highest 5-year OS and EFS rates was HR + HER2- (86.0% and 85.3%, respectively), while the TNBC phenotype had the worst survival rates (66.7% and 65.3%, respectively) (p < 0.05) (Table 6, Figs 6-9). According to stage, the OS and EFS were: stage I 89.4% and 89.9%; stage II, 88.1% and 80.0%; stage III, 72.4% and 59.2%; stage IV 47.1% and 41.2%; respectively (p < 0.001) (Table 6, Figs 10 and 11).

**Table 6:** Overall survival rates of Latin-Hispanic patients with special histological subtypes of breast cancer.

Results	Overall Survival				Event-free Survival			
	3y (%)	5y (%)	7y (%)	P value	3y (%)	5y (%)	7y (%)	P value
All population	88.7	82.1	76.9					
Histologic subtype				0.001				<0.001
Lobular	88.0	78.9	73.1		88.0	78.9	67.5	
Cribriform	100.0	100.0	93.8		100.0	100.0	93.8	
Apocrine	87.9	78.8	66.4		87.9	75.8	66.4	
Metaplastic	57.7	53.8	45.6		57.7	53.8	27.3	
Mucinous	93.3	88.3	86.6		93.3	88.3	82.6	
Neuroendocrine	100.0	75.0	75.0		100.0	75.0	75.0	
Medullary	72.2	72.2	72.2		72.2	72.2	66.7	
Papillary	93.3	90.0	84.7		93.3	90.0	80.1	
Micropapillary	89.5	78.9	69.1		89.5	73.7	40.5	
Tubular	95.2	95.2	79.4		95.2	90.2	83.8	
Mixed	90.9	88.6	83.8		90.9	88.6	79.0	
Others	100.0	77.8	77.8		88.9	77.8	83.83	
Phenotype								
HR + HER2-	92.8	86.0	81.4	0.002	92.3	85.3	75.9	<0.001
HR + HER2+	92.9	87.5	82.0		90.0	82.5	69.1	
HR- HER2+	84.0	76.0	76.0		87.5	75.0	75.0	
Triple negative	75.6	66.7	59.9		73.3	65.3	50.7	
Initial AJCC Stage				<0.001				<0.001
I	95.6	89.4	83.3		95.1	89.9	79.8	
II	92.7	88.1	83.6		89.2	80.0	70.0	
III	82.1	72.4	66.5		69.7	59.2	46.9	
IV	52.9	47.1	47.1		52.9	41.2	17.6	

AJCC: American Joint Committee on Cancer

Source: Authors’ own work.

**Fig 2:**
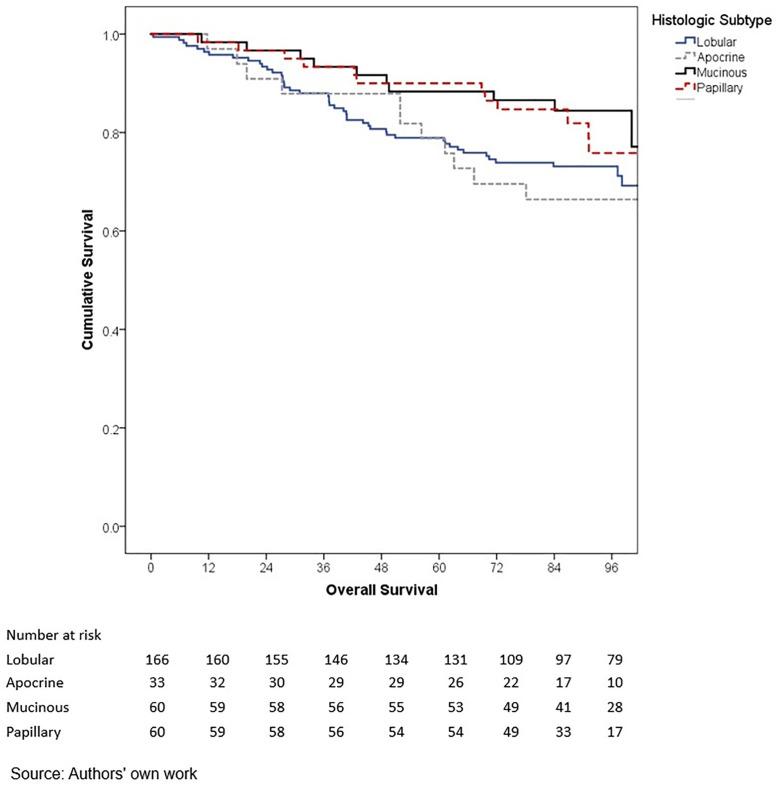
Kaplan-Meier curves of overall survival of Hispanic Latino patients with special histologic breast cancer subtypes according to the histologic subtype: lobular, apocrine, mucinous, papillary.

**Fig 3:**
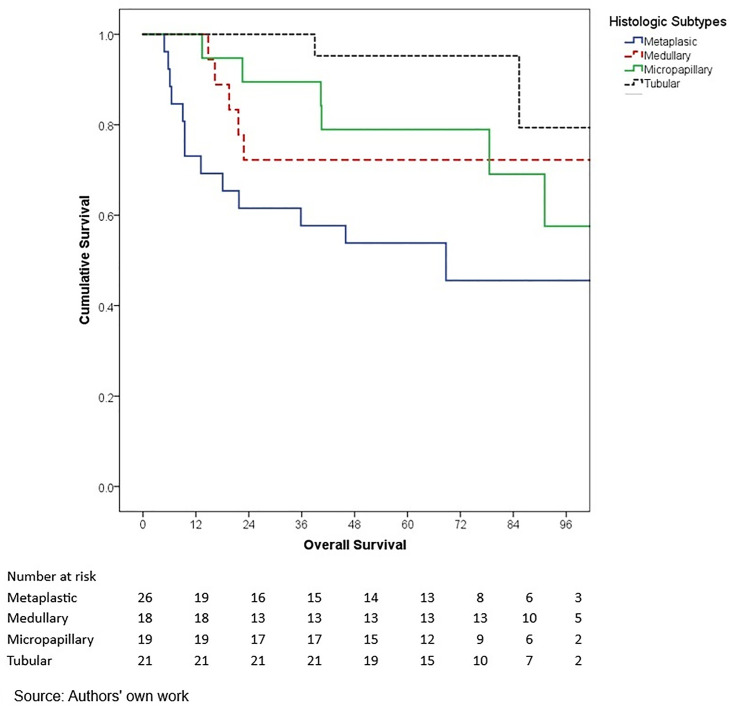
Kaplan-Meier curves of overall survival of Hispanic Latino patients with special histologic breast cancer subtypes according to the histologic subtype: metaplastic, medullary, micropapillary, tubular.

**Fig 4:**
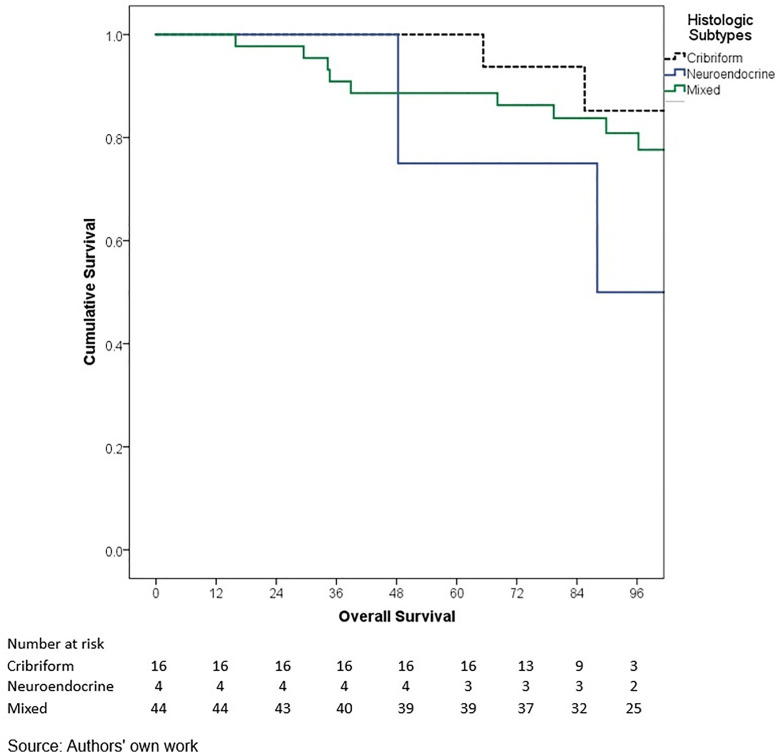
Kaplan-Meier curves of overall survival of Hispanic Latino patients with special histologic breast cancer subtypes according to the histologic subtype: cribriform, neuroendocrine, mixed.

**Fig 5:**
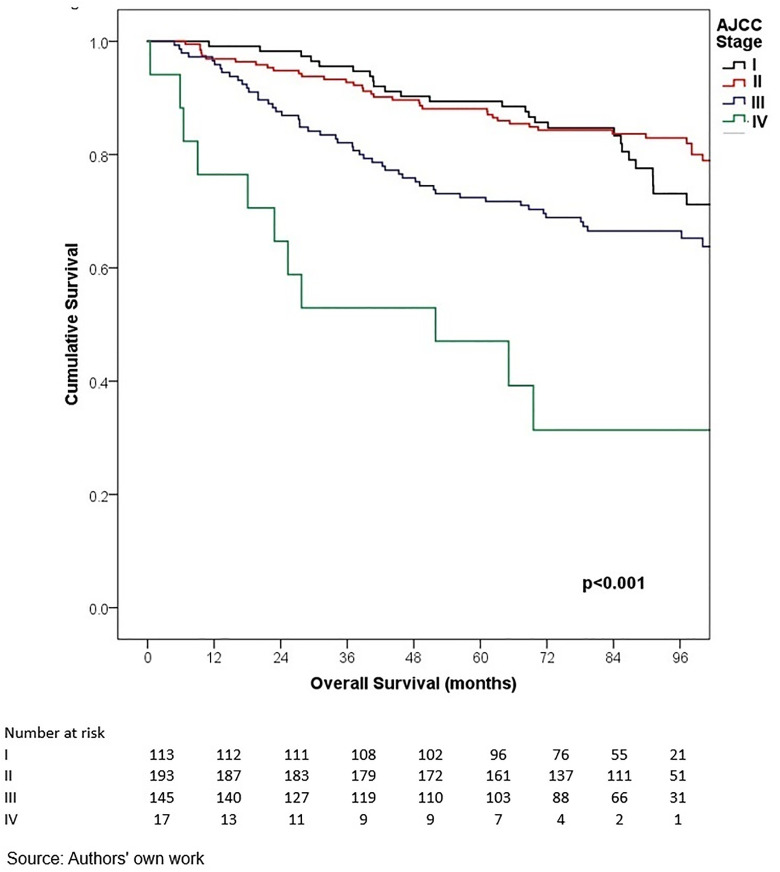
Kaplan-Meier curves of overall survival of Hispanic Latino patients with special histologic breast cancer subtypes according to the American Joint Committee on Cancer stage.

**Fig 6:**
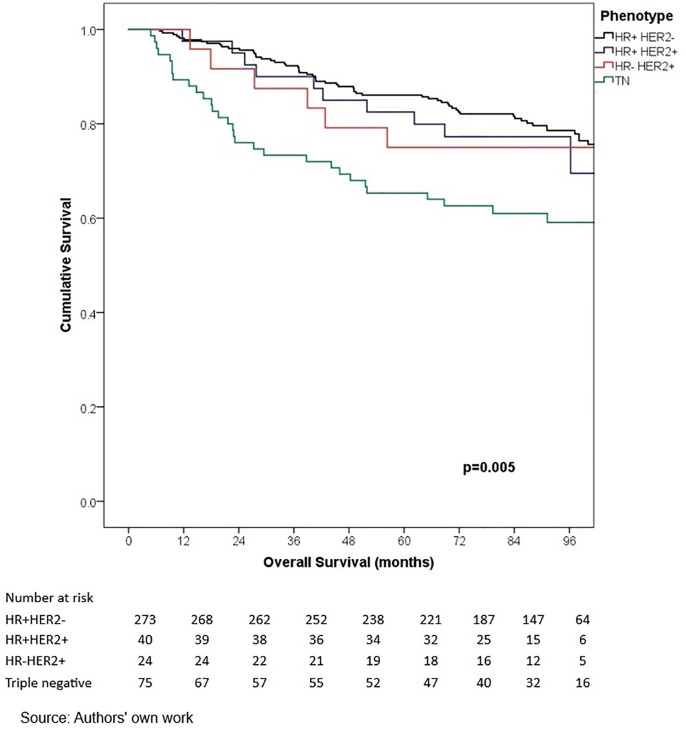
Kaplan-Meier curves of overall survival of Hispanic Latino patients with special histologic breast cancer subtypes according to the immunohistochemical phenotype.

**Fig 7:**
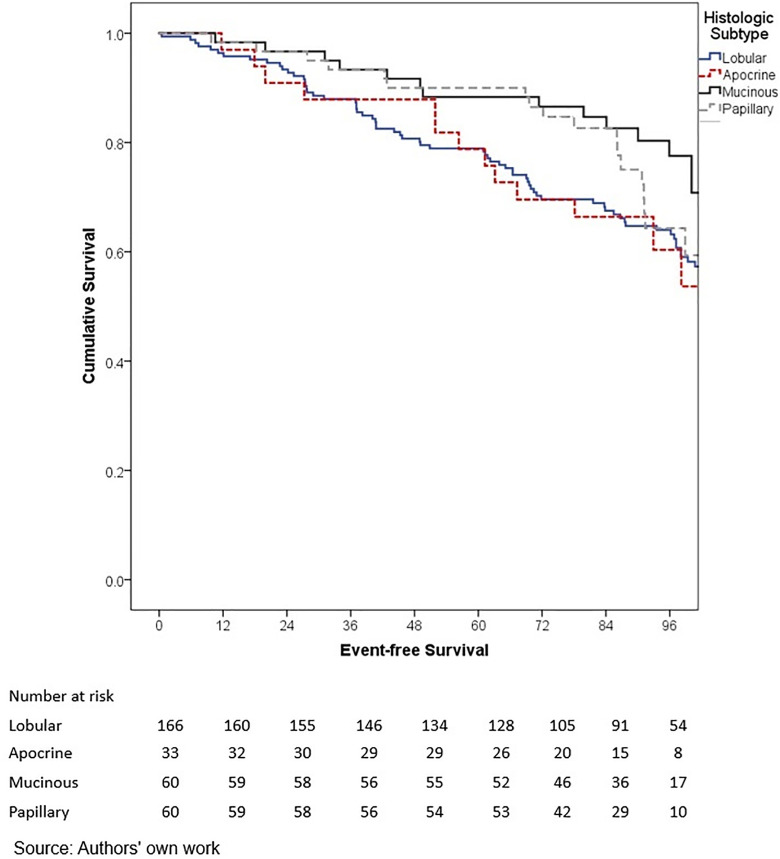
Kaplan-Meier curves of event-free survival of Hispanic Latino patients with special histologic breast cancer subtypes according to the histologic subtype: lobular, apocrine, mucinous, papillary.

**Fig 8:**
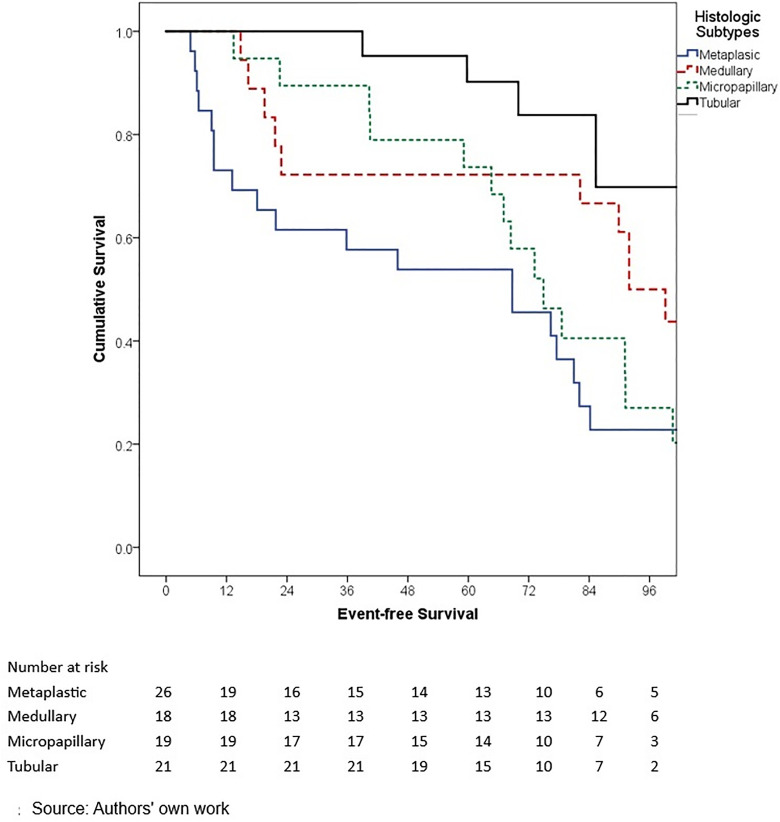
Kaplan-Meier curves of event-free survival of Hispanic Latino patients with special histologic breast cancer subtypes according to the histologic subtype: metaplastic, medullary, micropapillary, tubular.

**Fig 9:**
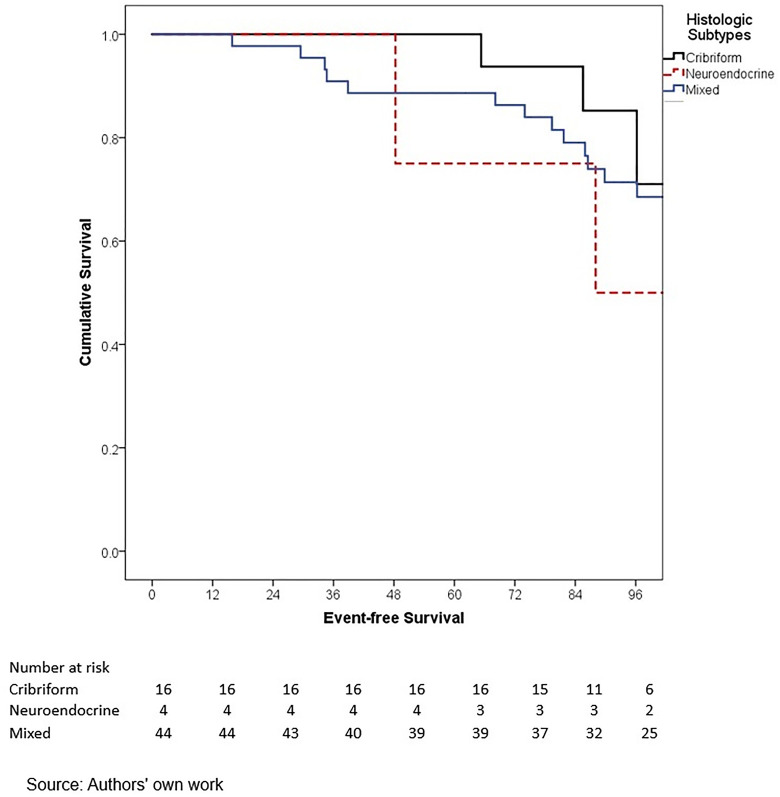
Kaplan-Meier curves of event-free survival of Hispanic Latino patients with special histologic breast cancer subtypes according to the histologic subtype: cribriform, neuroendocrine, mixed.

**Fig 10:**
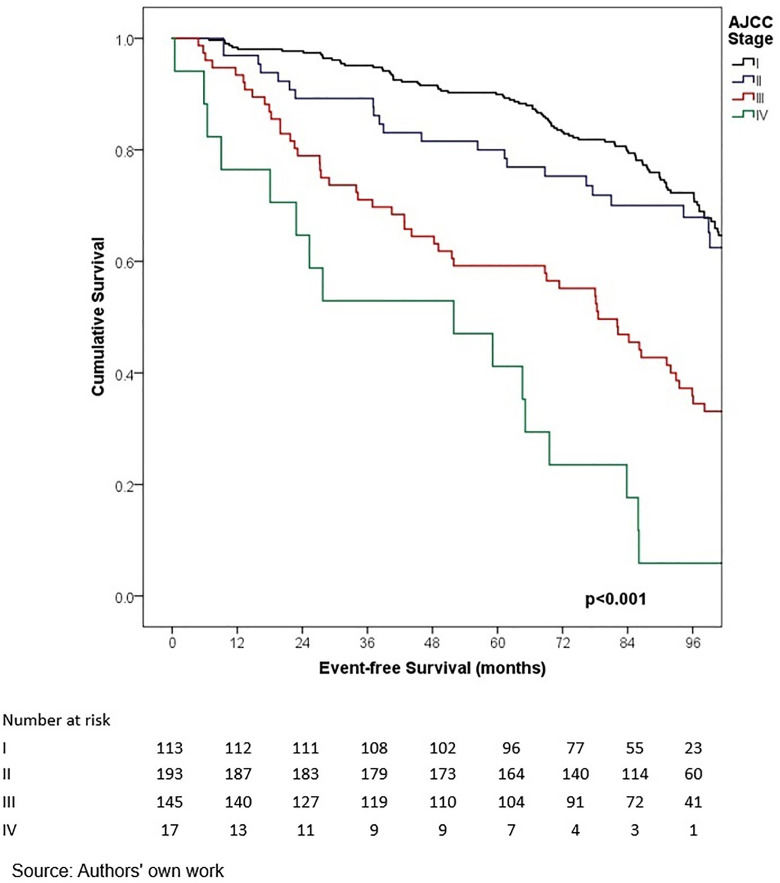
Kaplan-Meier curves of event-free survival of Hispanic Latino patients with special histologic breast cancer subtypes according to the American Joint Committee on Cancer stage.

**Fig 11:**
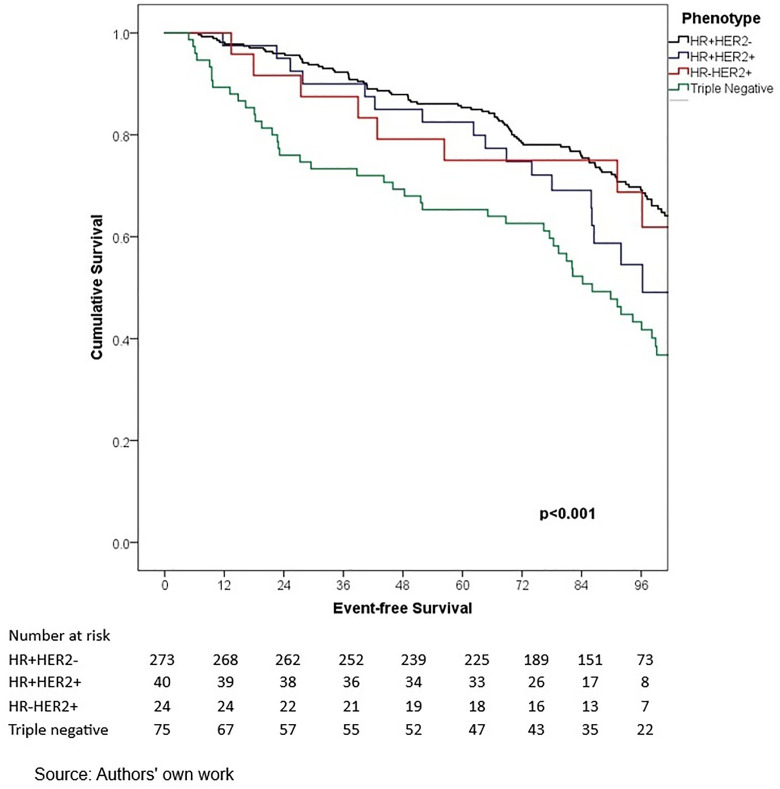
Kaplan-Meier curves of event-free survival of Hispanic Latino patients with special histologic breast cancer subtypes according to the American Joint Committee on Cancer stage.

## Discussion

The prevalence of histologic subtypes and phenotypes of breast cancer differ according to race and geographic location. We present one of the largest cohorts of Hispanic Latino patients with special histologic subtypes of breast cancer with unique characteristics and outcomes. Within this retrospective analysis of patients, lobular carcinoma was found to be the most frequent subtype with a prevalence of 35%, followed by mucinous, papillary, mixed, and apocrine variants. Most patients were diagnosed in stage II (40.7%), with 15.7% of TNBC. Despite half of the population receiving neoadjuvant treatment, surgical management consisted largely of mastectomy. While cribriform histology showed the most favorable outcomes, metaplastic carcinoma demonstrated poorer survival rates.

In regard to subtypes, we concluded that the most common subtype was lobular followed by mucinous and papillary. This in accordance with global reports [13,14] in which lobular carcinoma is indeed the most common amongst the special histological subtypes. In our sample, 5.8% of the patients had special histological subtypes, which is similar to a previous report in a Hispanic population [15], but significantly lower than other NHW and African-American (AA) populations with a prevalence of 5–15% [16]. In addition, a previous study reported that the incidence of these tumors increased with age, especially lobular breast cancer in which the frequency increases and reaches its peak at age 50 [17].

Although all the other subtypes represent less than 1% of the total breast cancer population, differences were found on comparison of NHW and Latin populations [18]. Subtypes such as mucinous (12.7% vs. 2%), papillary (12.5% vs. 1–2%), and apocrine (6.9% vs. < 4%) tumors were relatively more frequent, whereas mixed (9% vs. 25%), metaplastic (5.4% vs. < 1%), medullary (3.8% vs. 1–7%), cribriform (3.3% vs. 0.8–3.5%), and neuroendocrine (0.5% vs. 2–5%) carcinomas were less common [16,19,20]. These differences suggest that ethnicity may influence the prevalence of specific histological subtypes and could, therefore, affect tumor behavior, prognosis, and response to therapy.

The phenotype profile also varies according to region and race. While the most common immunohistochemical phenotype has been reported to be HR + HER2-, similar to our findings, the prevalence of TNBC differs among regions and populations. Previous literature showed that the prevalence of TNBC in Peru is 21.3% [21], which is within the upper range compared to other Latin populations with 12–24% [22], and is higher than that described in NHW women [5], and similar but lower than that of AA (15–30%) [23]. In our cohort, the lower frequency of TNBC and HER2 + tumors among the special histologic subtypes suggests that tumor biology and subtype composition may play an important role in the modulation of the expected regional or ethnic patterns of phenotypic expression [21,24]. Therefore this intersection of the prevalence of biology and population-specific subtype highlights the relevance of considering both histology and ethnic factors when evaluating disease prognosis and establishing treatment strategies [18,25].

Over 70% of patients had a ki67 of >20% which indicates a moderate proliferation rate. Of note, we found that elevated rates of ki67 persisted even after neoadjuvant chemotherapy. This phenomena has previously been described in lobular carcinomas, as they are less chemosensitive [26], resulting in lower rates of pathological response after chemotherapy, which is manifested by persistently high proliferation rates as well as lower rates of breast conserving surgery [27].

As reported in a prior meta-analysis, favorable outcomes of mucinous and papillary subtypes are largely due to intrinsic biology rather than response to chemotherapy. However, lobular and metaplastic tumors remain less responsive to systemic therapy and require surgical management [7,28,29]. This underscores the need to individualize the most adequate treatment for each patient according to their subtype, phenotype, and stage from a multidisciplinary point of view to optimize and offer the best treatment and obtain the best outcomes [30].

In relation to disease staging, most patients were found to have stage II followed by stage III tumors. Earlier stages were seen among this cohort compared to previous studies [21], which may be the result of the positive impact of multiple health campaigns such as the Plan Esperanza [31]. Lobular and metaplastic carcinomas often present at advanced stages due to diffuse or aggressive growth patterns, while mucinous tumors can be missed on physical examination

In terms of outcomes, 12.7% of patients presented distant recurrence as opposed to 4.7% developing locoregional relapse. Moreover, on assessing outcomes in lobular breast carcinoma, developed countries and studies based on NHW populations showed that the OS and event-free survival rates were 85% and 84%, respectively, while our results showed 79% and 79%, respectively. This finding might be explained by the higher stages at diagnosis among our patients and the higher frequency of TNBC [32,33] and limited availability of immunotherapy regimens. Nevertheless, when comparing only special subtypes to other different populations, the OS rates tended to show similar trends [7,34]. Lobular, cribriform, mucinous, and papillary carcinomas are all known to exhibit a lower rate of lymph node metastasis, and lobular carcinomas have less significant invasive and metastatic capabilities compared to ductal carcinoma, further explaining favorable outcomes [35]. Regarding mucinous and papillary tumors, the existing literature supports their low probability of relapse and in contrast to metaplastic tumors that display higher recurrence rates due to their aggressive nature [20,36]. In this respect, Zein *et al.* compared TNBC and metaplastic tumors and found that the latter displayed higher recurrence rates and a worse prognosis than TNBC [37]. Moreover, mucinous tumors tend to have lower recurrence rates due to reduced lymph node metastasis [38], hence ensuring a more favorable prognosis as well. Papillary tumors also present more favorable outcomes due to their lower histological grade as well as less aggressive features [7].

In terms of therapeutic characteristics, nearly 49% and 31% received neoadjuvant chemotherapy and hormonal therapy, respectively. Nowadays, neoadjuvant therapies play an important role in the management of breast cancer and differ across histological subtypes. Prior literature demonstrated that lobular carcinoma has a lower chemosensitivity compared to ductal carcinoma, as reflected in our study in which only one third of lobular tumors received neoadjuvant chemotherapy and approximately 50% received neoadjuvant endocrine therapy [28,39]. On the other hand, aggressive tumors, such as medullary, apocrine, and micropapillary carcinomas, present greater response to neoadjuvant chemotherapy and were therefore administered to more than 80% of our study population [39,40]. Favorable histologies such as tubular and mucinous, less frequently receive neoadjuvant chemotherapy but are administered higher rates of neoadjuvant endocrine therapy, which is consistent with their phenotypes and biologic behavior [41]. Patients with cribriform carcinoma underwent a mixed approach, with 62% of these patients receiving neoadjuvant chemotherapy and 37% neoadjuvant hormonal therapy. The heterogeneity among our population and across the subtypes underline treatment responsiveness and clinical decision-making tailored to each tumor behavior.

Our findings may have several potential applications in medical practice. First, targeted screening highlights the need to develop initiatives of community-level awareness and expand access to early detection services in Hispanic-Latino populations [42]. Second, they support the use of subtype-driven treatment strategies detecting the subtypes with the most favorable and poorest outcomes to guide risk-adjusted treatment planning with treatment de-escalation or more aggressive management. Third, these results can shape region-specific clinical guidelines, providing evidence tailored to this specific population. Fourth, they serve as a baseline for future multicenter studies [43]. Future research should aim to validate the subtype-specific survival patterns in larger multicentric cohorts and explore relevant biological markers and socioeconomic determinants. Moreover, future confirmatory studies based on our findings can serve as groundwork for personalized treatment planning and tailored management guidelines.

Another relevant area in which the results presented may be useful is that of developing artificial intelligence and a neural network-based diagnostic tool. The integration of clinical, pathological, and histological subtypes, and biomarker profiles could assist in the design of AI models as a tool for pathologists and oncologists to improve the diagnostic accuracy of rare and morphologically challenging special histologic subtypes. This tool could of great interest in community, private, and university hospitals [44,45]. These advances could potentially reduce the misdiagnosis and improve outcomes among Hispanic/Latino populations.

Despite the 6-year time period of this study, it has some limitations. Its retrospective design is intrinsically subjected to information bias, such as the fact that some clinical, pathological, and therapeutic characteristics collected from medical records were incomplete. Some patients were lost to follow-up and excluded from the analysis and this could have had an impact on the results. No BRCA1/2 or other molecular tests were available as these are not covered by the national insurance. Some patients could not be classified according to their immunohistochemical phenotypes as FISH was not performed to differentiate their HER2 receptor expression. However, this was a single institution study performed in a referral cancer institute which included all the population available, and no sample was used, and is therefore representative of the Peruvian population, Nonetheless, the present results should be interpreted with caution if extrapolated to other populations. The limited number of cases in certain special histologic subtypes (cribriform N = 16, neuroendocrine N = 4), restrict statistical power and require that outcomes for these groups be interpreted with caution. Furthermore, due to the relatively small number of patients within each special histological subtype group, performing an adjusted Cox proportional hazards model was not feasible. Therefore, our findings should be considered hypothesis-generating and may serve as a foundation for future research. Larger prospective multicentric studies are needed to validate our results, allow for more robust statistical analysis, and generate results that can be generalized to other countries and broader populations.

In conclusion, the frequency of special histologic subtypes of breast cancer in Hispanic Latino patients is unique. The most frequent subtype was lobular, followed by mucinous, papillary, mixed, and apocrine invasive carcinoma. More advanced stages were found compared to other developed countries, but similar or earlier compared to developing countries and previous studies from similar regions. The prevalence of TNBC was higher than the NHW population, but within the lower range of Hispanic populations. The Latin population with certain histological subtypes presented worse OS rates.

## Supporting information

S1 File Deidentified Excel dataset containing clinical and pathologic information on Hispanic/Latino patients diagnosed with special histologic breast cancer subtypes. (XLSX)

S1 Table Clinicopathological and therapeutic characteristics of Latin-Hispanic patients with breast cancer at diagnosis according to special histologic subtype. (DOCX)
