# Supplementary material for: Special histological subtypes of breast cancer in a Hispanic Latino population
Source: PLoS One. 2025 Oct 3;20(10):e0333139. doi: 10.1371/journal.pone.0333139 (PMC12494292; doi:10.1371/journal.pone.0333139)
Supplement: S1 Table — (DOCX) [file pone.0333139.s002.docx]

**Supplementary Table 1.** Clinicopathological and therapeutic characteristics of Latin-Hispanic patients with breast cancer at diagnosis according to special histologic subtype.

| Characteristics | Lobular  N=167 | Cribriform  N=16 | Apocrine  N=33 | Metaplastic  N=26 | Mucinous  N=61 | Neuroendocrine  N=4 | Medullary  N=18 | Papillary  N=60 | Micropapillary  N=20 | Tubular  N=21 | Mixed  44 | P value |
| --- | --- | --- | --- | --- | --- | --- | --- | --- | --- | --- | --- | --- |
| **Clinicopathological** |  |  |  |  |  |  |  |  |  |  |  |  |
| Initial T Stage |  |  |  |  |  |  |  |  |  |  |  |  |
| 0-2 | 118 (70.7) | 13 (81.3) | 12 (36.4) | 9 (34.6) | 45 (73.8) | 2 (50.0) | 7 (38.9) | 44 (75.9) | 10 (50.0) | 20 (95.2) | 32 (72.7) | <0.001 ^a^ |
| 3-4 | 49 (29.3) | 3 (18.8) | 21 (63.6) | 17 (65.4) | 16 (26.2) | 2 (50.0) | 11 (61.1) | 14 (24.1) | 10 (50.0) | 1 (4.8) | 12 (27.3) |  |
| Initial N Stage |  |  |  |  |  |  |  |  |  |  |  |  |
| 0 | 110 (65.9) | 13 (81.3) | 14 (42.4) | 14 (53.8) | 43 (70.5) | 3 (75.0) | 10 (55.6) | 36 (62.1) | 5 (25.0) | 19 (90.5) | 19 (44.2) | 0.037 ^a^ |
| 1-3 | 57 (34.1) | 3 (18.8) | 19 (57.6) | 12 (46.2) | 18 (29.5) | 1 (25.0) | 8 (44.4) | 22 (37.9) | 15 (75.0) | 2 (9.5) | 24 (55.8) |  |
| Initial M1 stage | 5 (3.0) | 0 (0.0) | 2 (6.1) | 4 (15.4) | 0 (0.0) | 0 (0.0) | 1 (5.6) | 3 (5.2) | 2 (10.0) | 0 (0.0) | 1 (2.3) | 0.120 ^a^ |
| **Therapeutic characteristics** |  |  |  |  |  |  |  |  |  |  |  |  |
| Neoadjuvant chemotherapy | 55 (32.9) | 10 (62.5) | 28 (84.8) | 14 (53.8) | 21 (34.4) | 3 (75.0) | 15 (83.3) | 31 (51.7) | 17 (85.0) | 5 (23.8) | 27 (61.4) | <0.001^b^ |
| Neoadjuvant radiotherapy | 6 (3.6) | 1 (6.3) | 3 (9.1) | 4 (15.4) | 3 (4.9) | 1 (25.0) | 4 (22.2) | 1 (1.7) | 2 (10.0) | 0 (0.0) | 2 (4.5) | 0.014 ^a^ |
| Neoadjuvant hormonal therapy | 51 (30.5) | 6 (37.5) | 14 (42.4) | 0 (0.0) | 21 (34.4) | 0 (0.0) | 2 (11.1) | 28 (46.7) | 10 (50.0) | 2 (9.5) | 16 (36.4) | 0.001 ^a^ |
| Neoadjuvant trastuzumab | 8 (4.8) | 5 (31.3) | 16 (48.5) | 1 (3.8) | 1 (25.0) | 3 (16.7) | 19 (31.7) | 9 (20.5) | 9 (45.0) | 3 (14.3) | 13 (26.0) | <0.001 ^a^ |
| Surgery |  |  |  |  |  |  |  |  |  |  |  |  |
| Breast conserving surgery | 38 (23.2) | 8 (50.0) | 4 (12.1) | 0 (0.0) | 23 (38.3) | 2 (50.0) | 4 (22.2) | 19 (33.9) | 4 (22.2) | 13 (61.9) | 15 (34.9) | 0.001 ^a^ |
| Mastectomy | 126 (76.8) | 8 (50.0) | 29 (87.9) | 26 (100.0) | 37 (61.7) | 2 (50.0) | 14 (77.8) | 37 (66.1) | 14 (77.8) | 8 (38.1) | 28 (65.1) |  |
| Missing | 3 | 0 | 0 | 0 | 1 | 0 | 0 | 4 | 2 | 0 | 1 |  |
| Adjuvant chemotherapy | 117 (70.1) | 14 (87.5) | 31 (93.9) | 21 (80.8) | 30 (49.2) | 4 (100.0) | 17 (94.4) | 39 (65.0) | 17 (85.0) | 14 (66.7) | 36 (81.8) | <0.001 ^b^ |
| Adjuvant radiotherapy | 114 (68.3) | 15 (93.8) | 27 (81.8) | 17 (65.4) | 44 (72.1) | 2 (50.0) | 14 (77.8) | 51 (85.0) | 17 (85.0) | 17 (81.0) | 40 (90.9) | 0.016 ^b^ |
| Adjuvant hormonal therapy | 152 (91.0) | 10 (62.5) | 16 (48.5) | 2 (7.7) | 60 (98.4) | 1 (25.0) | 2 (11.1) | 46 (76.7) | 16 (80.0) | 18 (85.7) | 32 (72.7) | <0.001 ^a^ |
| Adjuvant trastuzumab | 3 (1.8) | 5 (31.3) | 11 (33.3) | 1 (3.8) | 1 (1.6) | 1 (25.0) | 1 (5.6) | 7 (11.7) | 3 (15.0) | 5 (23.8) | 6 (13.6) | <0.001 ^a^ |

^a^ chi-square test

^b^ Fisher’s exact test

Source: Authors’ own work.
